# Addressing food system determinants of health inequalities in urban environments: learnings from the FoodSEqual and FoodSEqual-Health projects

**DOI:** 10.1098/rstb.2024.0150

**Published:** 2025-09-18

**Authors:** Carol Wagstaff, Clare Pettinger, Clare Relton, Katerina Psarikidou, Elaine Swan, Lisa Methven, Gunter Kuhnle, Rachel McCloy, Sally Lloyd-Evans, Michelle Thomas, Manik Puranik, Martin Chadwick, Lorna Zischka, Rachel Smith, Hannah Gardiner, Louise Hunt, Jiang Pan, Jane Bradbeer, Rachel Sutton, Barbara Diouri, Emmaleigh Williams, Lisa Alloune, Patricia Bennett, Lisa Howard, Paridhi Garg, Yve Ashton, Joanne Hart, Sajna Miah, Shazna Hussein, Julie Yip, Shelley Taylor

**Affiliations:** ^1^ Department of Food and Nutritional Sciences, University of Reading - Whiteknights Campus, Reading, England, UK; ^2^ School of Health Professions, University of Plymouth Faculty of Health and Social Work, Plymouth, England, UK; ^3^ Science Policy Research Unit, University of Sussex, Brighton, UK; ^4^ Department of Management, University of Sussex, Brighton, UK; ^5^ School of Psychology and Clinical Language Sciences, University of Reading - Whiteknights Campus, Reading, UK; ^6^ Department of Geography and Environmental Sciences, University of Reading - Whiteknights Campus, Reading, UK; ^7^ Whitley Community Development Association, Reading, Berkshire, UK; ^8^ The Local Sustainable Food Places Partnership, Food Plymouth CIC, Plymouth, Devon, UK; ^9^ Womens' Environment Network, 20 Club Row, London, UK; ^10^ Brighton and Hove Food Partnership, Brighton, Sussex, UK

**Keywords:** food system, disadvantaged communities, community engagement, health inequalities

## Abstract

Diet is just one social determinant of health, with obesity and diet-related illness as leading causes of premature deaths and disability in the UK. People living in deprived areas of the UK are more likely to experience poor health outcomes, linked to many social determinants of health such as diet, housing, employment, education, power and discrimination and psycho-social factors such as isolation, quality of social network and perceived level of control. While socio-economic deprivation is a key risk for health inequalities, protected characteristics, vulnerable groups and geographical factors also indicate populations at risk of experiencing health inequalities. In this ‘opinion article’ we draw on our experiences in the Food Systems Equality (FoodSEqual) and FoodSEqual-Health research projects to share our methods of engaging residents from a range of population groups in areas of high deprivation in England, who are often under-represented in studies related to diet and health. We consider the contextual factors that shape the lived experience of residents, the contributions they can make when engaged in research and social development, and advocate for their inclusion in developments designed by voluntary sector organizations, local authorities and central government to improve the food systems they access. Finally, we consider the role of local food strategies and policy actions in developing healthy, sustainable and equitable food systems.

This article is part of the theme issue ‘Transforming terrestrial food systems for human and planetary health’.

## 1. Introduction

The food system is a multifaceted complex web of actors and organizations that are necessary for the production, transformation, distribution and retailing of food to the consumer across the food chain [1,2]. While the global food system produces enough calories to feed everyone on the planet [3] there are disparities in access (physically and economically) that impact overall diet quality within and between social groups in society. There is clear evidence of a socio-economic gradient in health and that diet is a modifiable risk factor for the development of non-communicable diet-related disease.

However, the food system, quality of the food and food security are part of a wider set of factors that contribute to health inequalities seen in our society. Health inequalities are defined as unfair and unjust health differences between population groups, which are directly or indirectly generated by social, economic and environmental factors and structurally influenced lifestyles [4]. These determinants of social inequities—and therefore health inequalities—are all amenable to change [5]. Population groups that are more likely to experience health inequalities are those with protected characteristics (e.g. a disability, age, race)those who are socio-economically deprived (e.g. the wider/social determinants of health), inclusion and vulnerable groups (e.g. Roma and Travellers, people experiencing homelessness) and people disadvantaged owing to geography (e.g. built and natural environment, coastal communities, levels of social connectedness). Often multiple forms of disadvantage overlap exacerbating health inequalities ([6] in National Institute of Health and Care Excellence report (see [6], accessed 27/11/2024).

**Figure 1.**
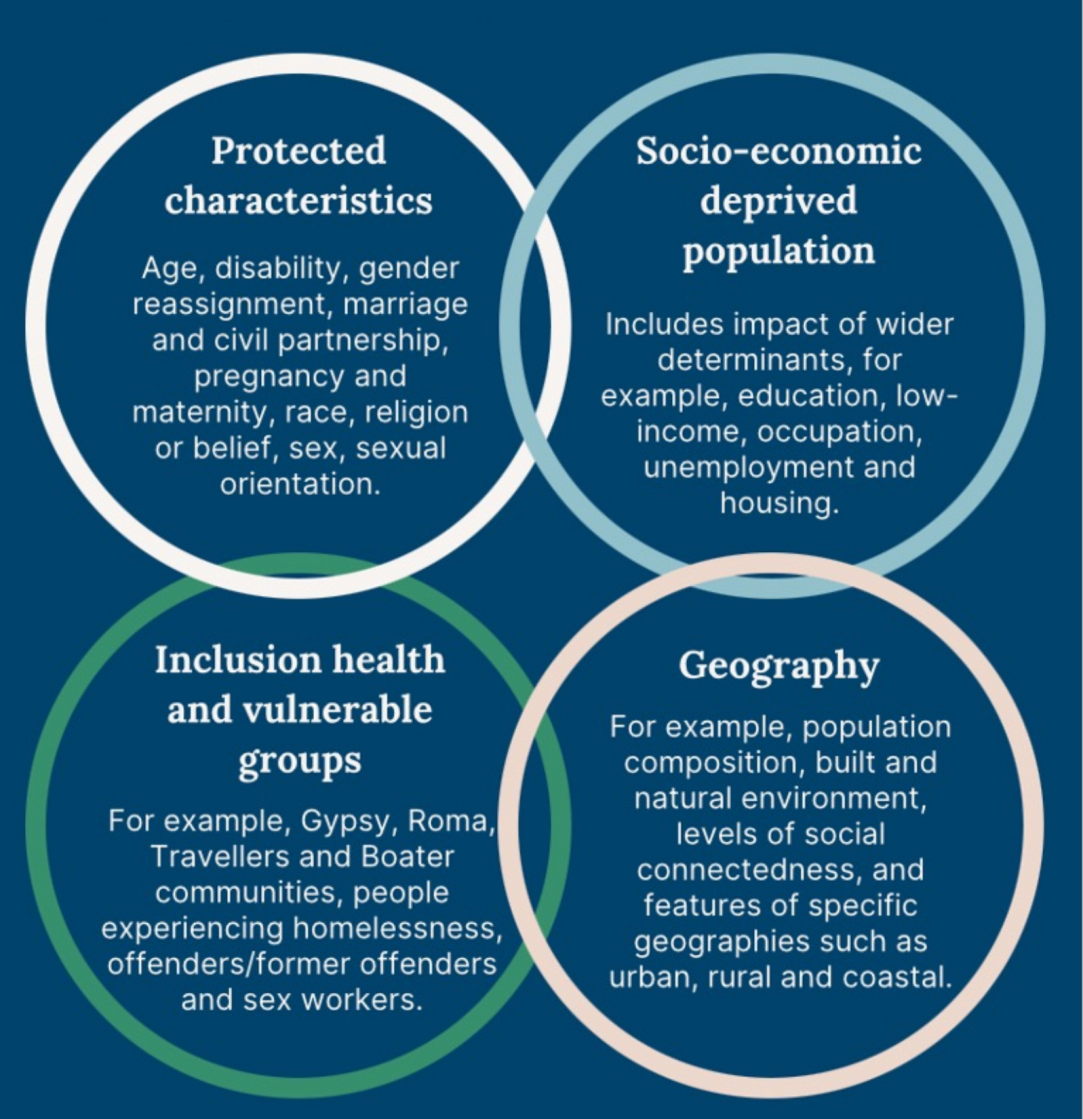
Population groups at risk of experiencing health inequalities. Reproduced from National Institute of Health and Care Excellence (https://www.nice.org.uk/about/what-we-do/nice-and-health-inequalities).

Wider/social determinants of health cross almost all departments of central and local government, including housing, transport, work and pensions, health and social care, education and economics. If residents are disadvantaged in one or more of these determinants (figure 1) they are likely to experience poor health outcomes and may also have poor access to healthcare [7,8]. It is therefore critical that health inequalities are not seen as the sole preserve of departments of public health, either at national or local level, and cross-departmental working is needed to overcome the siloed approach that characterizes our present system [9].

Urban environments frequently represent locations where material deprivation is concentrated and where the disparities between rich and poor become more obvious [10–12]. All types of household structures are impacted by deprivation. The current list of questions posed in the Family Resources Survey (2023−2024 onwards; Dept Work and Pensions, https://www.gov.uk/government/statistics/family-resources-survey-financial-year-2023-to-2024) includes asking people whether everyone in the family eats three meals a day and whether fresh fruit and/or vegetables are consumed by all family members every day as part of a set of indicators used to measure material deprivation in the UK. Urban place-based interventions bring opportunities to improve the social determinants of health by investing resources from local councils and non-governmental organisations. Although many such schemes have been initiated in a variety of countries there is limited evidence of lasting improvement [13–15].

Place-based interventions are defined as both (i) a particular area or place considered together with its inhabitants and (ii) the condition of sharing or having certain attitudes and interests in common. Place-based interventions aimed at addressing health inequalities offer an opportunity to harness the power of community involvement. Building community cohesion is about creating a sense of togetherness, which in the context of food initiatives is often cited as a lever that encourages people to engage in collective action, solidarity and community self-organization to pursue a common goal [16–20].

### (a) Multi-level action is required to be effective

Local government initiatives that are designed to address health inequalities proliferate across the UK. However, the resource invested often ends up creating supportive jobs for professionals and rarely tackles the underlying causes of, nor directly addresses, poverty. For example, the recent Local Government Association report on Inclusion Health [21] describes 10 case studies that were resourced by local councils, of which six had a strong element of surveying and measuring the problem being studied, rather than providing interventions. Poverty is often at the root of health inequalities, and interventions taking a ‘cash-first’ approach or that are focused on price are the most likely to succeed [22,23].

Many state and voluntary sector organizations are keen to fund local projects that adopt an ‘asset-based’ approach to changing individual behaviours and lifestyle choices without addressing their structural determinants [24]. However, the lack of overarching social and capital infrastructure limits communities’ capacity to drive more equitable systems, which depend upon supporting infrastructure [25]. Behaviour change observed in participants during interventions is likely to be unsustainable in the long term unless the structural factors that give context to their choice are also changed [26]. In this paper, and existing FoodSEqual publications from Hunt *et al*. [25] and Briazu *et al.* [26], we therefore call for multi-level interventions on the wider social determinants of health that integrate the individual-, community- and macro-level actions.

United Kingdom Research and Innovation (UKRI)’s ‘Transforming the UK Food Systems’ (TUKFS) Strategic Priority Fund has supported substantial projects that have been able to engage individuals, communities, voluntary sector organizations, local authorities and central government departments in a way that has enabled multi-level action. The unifying aim has been to facilitate the development of an economically, environmentally and socially sustainable food system that allows citizens to access a healthy diet, which is a crucial social determinant of health equity. FoodSEqual (Food Systems Equality) is one of four large projects supported in the first round of TUKFS funding and subsequently through an extra funding stream directed specifically at dealing with food system-related health inequalities. FoodSEqual is a complex transdisciplinary multiple-mixed methods research programme focused on residents from four disadvantaged communities, which aims to enable improved access to nutritious, sustainable, culturally appropriate, affordable and healthy food that people want to eat. In our research, we use ‘community’ to indicate a geographical place, but also the sense of connectedness that exists between people who have shared beliefs, values or experiences. Our research uses a ‘co-production’ approach to develop solutions that meet the needs of diverse stakeholders including residents, businesses and local authorities [27]. We set out to develop methods and methodologies for changing a food system in a local area that would not only work for the four culturally diverse urban communities with which we work closely (Whitleigh in Plymouth, Whitley in Reading, Brighton & Hove and Tower Hamlets), but that are translatable to other communities around the UK. From the outset, we recognized that the solutions appropriate for a particular place were likely to be different for each community, but we aimed to develop a unified methodology that enabled residents to articulate their ambitions and to be supported in achieving them. Our methodology provides insight for policy-makers in the UK who have recently developed a new focus on neighbourhoods in national policy development (through the launch of the Independent Commission on Neighbourhoods; https://www.neighbourhoodscommission.org.uk/) as a means of addressing multiple disadvantage, regenerating communities and enabling government to achieve its mission objectives.

### (b) Research gap and objectives

In this paper, we tackle the gap that exists in how to support residents, voluntary sector organizations, local authorities and central government to work effectively together with a shared aim of improving the food system for residents of disadvantaged communities. Bringing people together who have historically been excluded from policy development and decision-making has been challenging for the FoodSEqual team and has required researchers and residents to overcome practical difficulties and preconceptions so that we could carry out research together effectively. The experiences of the FoodSEqual team, working in four culturally diverse communities with food industry partners of all sizes, voluntary sector organizations, local authorities and central government departments, have provided us with insight on how to make positive change to food systems in the UK that are accessed by residents from disadvantaged communities. Residents are playing a pivotal role in our research process, working alongside academics, industry and civil organizations, to co-create innovative solutions in an environment where they have a voice in the room and are able to share their ‘lived experiences’ [28,29]. Here we describe our vision for community engagement that enables localized responses to a centralized food policy. Our approaches have enabled residents to become active food citizens [30] who could, in some cases, use their agency to positively influence their own and others’ lives.

The methods developed in FoodSEqual underpinned the work we have undertaken more recently in FoodSEqual-Health, which more specifically addresses health inequalities in the food system, basing interventions at sites in Whitleigh in Plymouth and Whitley in Reading. We were able to move faster and engage more deeply with residents during FoodSEqual-Health because we had already established relationships and ways of working in the original FoodSEqual project.

In this paper, we draw on our methods developed in FoodSEqual and FoodSEqual-Health to:

— Articulate the importance of contextualizing the food systems and food lives that impact on resident groups in disadvantaged areas.
— Exemplify effective community engagement approaches, which have successfully involved place-based research with residents traditionally categorized as ‘hard to reach’.
— Advise on managing partnerships and power dynamics between different groups who have a common goal (wanting to change a food system) but individual perspectives and priorities.
— Discuss how to ensure that residents' voices are heard and acted upon, alongside enabling people to imagine what their future food system might look like.

## 2. Methods

### (a) Community food researchers used throughout FoodSEqual and FoodSEqual-Health

National datasets such as the National Diet and Nutrition Survey (NDNS; https://www.gov.uk/government/collections/national-diet-and-nutrition-survey) and The Living Costs and Food Survey (LCF; https://www.ons.gov.uk/surveys/informationforhouseholdsandindividuals/householdandindividualsurveys/livingcostsandfoodsurvey), describe disadvantage in numbers, such as the types and volumes of food purchases, but hide the context within which decisions about daily life are taken by individuals in disadvantaged communities [25,31,32]. In FoodSEqual, we have recruited and trained individuals who live in the communities we work in and who are knowledgeable, skilled and confident to deliver research activities that engage local residentsto be Community Food Researchers (CFRs) [33]. The CFRs and academic teams agree on the problem to be solved, the questions that need asking, the language and methods to be used. The knowledge of cultural norms in each area and lived experience of the CFRs were critical in shaping the research design so that residents were more likely to engage.

### (b) Using a range of approaches across FoodSEqual and FoodSEqual-Health

A wide range of quantitative, qualitative and participatory creative methods have been explored to date across the FoodSEqual and FoodSEqual-Health research projects. Briefly, they include interviews (structured and unstructured), focus groups (typically using participatory methods such as visioning, storyboarding, collage, zine (do-it-yourself booklets or maps) and drawing), workshops (such as film making and co-design with school students), creative ethnography methods (such as photo- and video-diaries, cookalongs) and quantitative methods (such as questionnaires, 24 h dietary recall, biomarker assessment, real-world choice experiments and eye-tracking). The biomarker study (NCT05907889) uses objective markers for dietary assessment and encompasses quantitative assessment methods. Qualitative analysis includes both inductive and deductive extraction of themes (for example from interview transcripts). Resulting themes were evaluated within the communities, either through the CFRs [33] or with the community members using participatory analysis. Quantitative or semi-quantitative data were summarized and analysed through a range of statistical methods, from descriptive statistics through to mixed models. Wherever possible, we have taken approaches that allow participants (whether from industry or community residents) to connect and share their experiences in their own words. Our methods were ethically approved by institutional Research Ethics Committees and can be accessed at [34].

### (c) Synthesis of community engagement messages from FoodSEqual

Common across all elements of the FoodSEqual project is ‘community engagement’. Each site had different demographic characteristics and different prior histories of engagement with research and/or food projects. In this paper, we sought to bring together our experiences so that other teams seeking to follow a similar journey can learn from us. To help us reflect concretely on our learning across our four sites, the lead author conducted interviews with the research teams represented by CFRs and academics in the Universities of Plymouth, Sussex and Reading. The questions were co-developed by the lead authors, H. Gardiner (who is undertaking a PhD related to this area) and C. Pettinger in order to identify key themes across FoodSEqual and to highlight points of contrast. The interviews were semi-structured, with responses to the following questions obtained from each research team in the four geographical areas participating in FoodSEqual:

— What relationships were in place between the academic base, voluntary community and social enterprise (VCSE) sector and local residents in your area before FoodSEqual started? Were these direct relationships or mediated via another organization e.g. food partnership, community association? E.g. some places already had community researchers; some universities already had collaborations with VCSE sector partners; some areas already had food partnerships.
— How has the relationship between the academic base, VCSE sector and residents developed during FoodSEqual? Consider who has engaged, trust, understanding, listening. What is different now compared with the start? What evidence do you have for any of these observed changes?
— What has the relationship between the academic base, VCSE sector and residents enabled you to ask? i.e. what were your research questions? Could you have done this without the relationships in place? Do you have any evidence that the quality/depth of answers or experiences shared by residents is influenced by the relationships?
— Please highlight any methods you used that you feel have been really effective. Why did they work so well? What was achieved?

The authors have drawn out common themes and highlighted points of difference in §3b.

### (d) Fresh Street-Community: an intervention delivered through FoodSEqual-Health

Fresh Street (www.freshstreet.uk) is an area-based scheme that provides vouchers for residents that can be redeemed for fruit and vegetables in local outlets (market stalls, independent greengrocers), addressing both affordability and accessibility. The vouchers are provided to all households in an area without means testing. Fresh Street has previously been tested in urban areas of high deprivation in the UK (Barnsley, Sheffield, Doncaster, Bradford and Tower Hamlets) and evidence indicates that it leads to an increase in fresh fruit and vegetable consumption [35–37].

Fresh Street Community (https://www.freshstreet.uk/fresh-street-community/) is an ongoing version of the intervention that combines regular vouchers with community centre-based fruit and vegetable stalls or collection points. Fresh Street Community is running in both Whitley, Reading and Whitleigh, Plymouth, UK, under the banner of the FoodSEqual-Health project. Both areas represent some of the most deprived wards in the UK, using indices of multiple deprivation as a measure [38]. Full results of the intervention will be published when the research has concluded, but a protocol is included here, since the ‘community’ aspects of the intervention have driven some of the thinking in this paper.

In each location, streets were selected that were approximately equidistant from a community hub that is used as the base from which the intervention is executed. Approximately 300 households are represented in each location; of these, 150 households received £10-worth of Fresh Street vouchers per fortnight (providing economic access). These have been delivered directly to households' doors by the project team and they provide residents with economic and physical access to fruit and vegetables supplied by the local wholesalers (Tamar Fresh in Plymouth and CA Belcher in Reading). Households not receiving Fresh Street vouchers (control group) had enhanced physical, but not economic, access to fruit and vegetables.

In Plymouth a pre-order system was set up: Tamar Fresh made up the £5-value produce boxes according to what was fresh and in season each fortnight, plus a monthly market stall was initiated in the second half of the intervention, which increased resident engagement and voucher redemption. In Reading, a fruit and vegetable stall is run every Saturday morning from 09.30 to 11.30, staffed and organized by the Whitley Community Development Association (WCDA) and based at the South Reading Community Hub, which functions as their operational base. The hubs act as more than a point of transaction for the fruit and vegetables: all households in the intervention, whether in receipt of vouchers or not, are encouraged to engage with the health and social care services offered at the hub (at Reading these include NHS health checks, Citizens Advice clinics, Communicare consultations, social activities, a café). In Plymouth, adjunct social engagement activities were co-designed and delivered by CFRs to support the cooking, storage and consumption of fresh fruit and vegetables.

#### (i) Intervention evaluation within FoodSEqual-Health

Diet quality, fruit and vegetable intake, markers of health and well-being were measured at baseline and then at six-month (Reading) and 12-month (Plymouth and Reading) intervals after starting the Fresh Street Community intervention. The ‘Food and Wellbeing Chat’ covers diet quality, food affordability, well-being and 24 h dietary recall. It comprises a combination of INTAKE24 [39], plus selected questions from the Short Warwick-Edinburgh wellbeing survey [40], the NHS wellbeing survey [41] and the Food Standards Agency Food Affordability Survey [42]. Our local Reading NHS partners have shared anonymized data from health checks performed at the community centre where the fruit and vegetable stall is based, both at baseline and 12 months into the intervention, covering health indicators such as weight management, blood pressure and blood glucose levels, all of which are indicative of risks associated with diet-related illness such as cardiovascular disease and diabetes.

#### (ii) Process evaluation of FoodSEqual-Health

Methods developed for outcome and process evaluation of FoodSEqual-Health and Fresh Street-Community are based on a framework incorporating acceptability, fidelity and feasibility. The evaluation engages a range of stakeholders including citizens, fruit and vegetable wholesalers, healthcare professionals and local authority public health and community departments. It examines implementation (via interviews), mechanisms of impact (underpinned by the COM-B, which proposes that behavior (B) is influenced by three key components: Capability (C), Opportunity (O), and Motivation (M), and the Behaviour Chainge Wheel; [43]) and contextual factors (such as pre-existing relationships, other services in the area). We also consider barriers and enablers to the intervention using exploratory qualitative mixed-methods and informed by ‘co-production’ with community researchers [27] in three phases: (i) literature review identifying enablers and barriers on fruit and vegetable voucher schemes; (ii) semi-structured interviews and focus groups to ‘validate’ literature review (research team and CFRs from both sites); (iii) comprehensive analysis (including CFRs) of identified enablers and barriers to inform factors and accompanying explanatory narratives [44]. Observations of community engagement processes were also collated by CFRs.

These evaluations are ongoing and will be separately published when concluded. They will help to articulate how preventative action can mitigate the rising cost of healthcare if dietary inequalities persist. Translation of our findings into Quality Adjusted Life Years (QALYs), potential savings in NHS care etc. through health economics modelling will ultimately inform policy development and public investment of funds.

## 3. Insights from FoodSEqual and FoodSEqual-Health research

Researchers and policy-influencers alike must deal with the challenge of residents feeling jaded and worn down by decades of unfulfilled promises or ineffective place-based programmes that fail to deliver tangible improvements to their lives [45]. Residents are often strongly engaged with local challenges and resistant to ‘outsiders’ coming in to ‘do to’ them [46]. Many community groups feel they are over-researched, leading to feelings of being used to measure the problem without seeing any meaningful change in their own lives [47]. Researchers seeking to conduct research with residents in the future should find ways of working that directly support residents and communities to improve their situations as part of the research process—as manifested by e.g. Lloyd-Evans *et al.* [48]—rather than adding to the stress on those involved.

**Figure 2.**
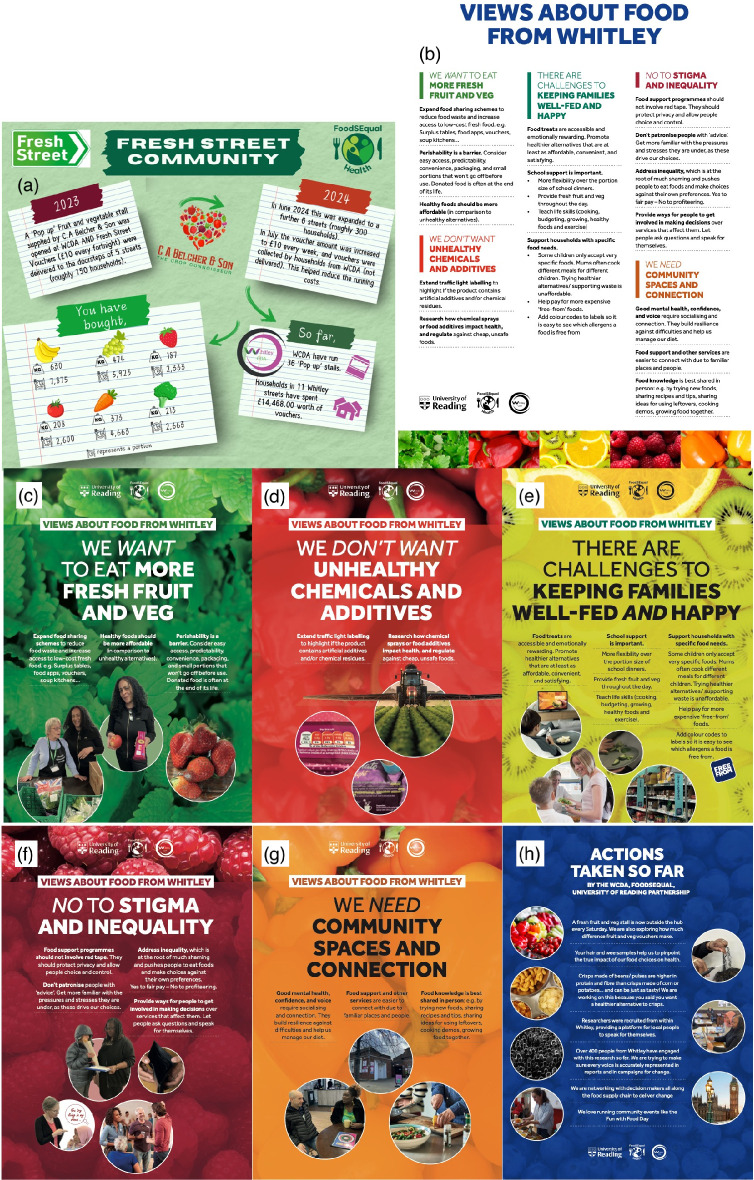
Conveying messages to residents on (a) the scale of their contribution to the intervention and (b*–*h) the messages heard by researchers as a consequence of the data collected in the intervention. Examples are provided from FoodSEqual research in Whitley, Reading. Original pdf files of the individual images are available in the electronic supplementary material.

Our research in FoodSEqual is some of the first to address food system inequalities by looking to residents and those at increased risk of health inequalities to understand their views, thoughts and opinions and to co-develop solutions. The place-based approach we have taken in FoodSEqual-Health built on the engagement we had developed in FoodSEqual, which enabled us to both do research and make a favourable impact on peoples’ lives. Critically, it has enabled residents to see some positive consequences for their communities in a relatively short space of time (https://research.reading.ac.uk/food-systems-equality/wp-content/uploads/sites/186/2024/11/FoodSEqual-report-FINAL.pdf). In this example, it was the provision of fruit and vegetables in areas classified as ‘food deserts’, coupled with vouchers that provide economic access to the fresh produce. Providing feedback to participants that illustrates the scale of their participation and details the collective messages that are being heard by researchers conveys to all those involved that the individual participants belong to a community-level project (figure 2). Our work has so far reached only a small number of residents relative to the total population of the Wards we are working in, but our methods pave the way for future work that would enable positive and enduring changes to food systems, community development and health equity in other areas.

### (a) Contextualizing determinants of health

FoodSEqual shaped the approach that we now advocate for community engagement aligned to transforming the food system. This section illustrates the importance of the benchmarking work undertaken in FoodSEqual to describe the ‘foodscape’ in our different communities and discovering the context in which people made choices about what they ate.

Quantitative datasets using dietary survey methodologies are the predominant source of information about UK adult diets [49] yet they may mis-represent diets in disadvantaged communities because sub-sample sizes are too small to be included in statistical analyses [31] and they fail to consider wider structural perspectives [32]. The first phase of FoodSEqual was to benchmark what people currently consumed in their diets, but, unlike the types of data collected for national datasets such as the National Diet and Nutrition Survey ([5] onwards), we were also determined to understand why people consumed what they do, describing the foodscape of people’s lives [50].

From the outset, our approach—which was underpinned by the evidence obtained by community researchers recruited from the population of residents on which the research is focused (https://research.reading.ac.uk/food-systems-equality/brighton-hove-community-researchers-their-journey-of-learning/)—provided insights that are missed in national data but that are critical to understanding lived experience of material, cultural and economic disadvantage. These ranged from understanding food consumed in a typical day, to understanding the drivers for these food choices and residents’ aspirations for change. For example, in more than one community participating in FoodSEqual research people expressed aspirations to increase their vegetable intake, although the aspirations varied between communities from greater access to locally grown culturally appropriate vegetables (https://research.reading.ac.uk/food-systems-equality/from-pot-to-polytunnel-how-a-collaboration-with-project-partner-tozer-seeds-may-produce-climate-hardy-seeds-for-locally-grown-bangladeshi-cultural-vegetables/) to more affordable local vegetables packed and distributed in a sustainable way (https://research.reading.ac.uk/food-systems-equality/community-food-research-in-brighton-and-hove-what-we-have-learnt/).

We had to consider the role of the eating environment and the influence that food has on social structure (and *vice versa*). People living in areas of high deprivation are often criticized by people from outside these communities for not consuming the cheapest foods, not cooking from scratch or not growing their own vegetables. FoodSEqual took the time to understand lived experience (https://research.reading.ac.uk/food-systems-equality/tower-hamlets-community-researchers-how-food-diary-research-works/) and residents told us about the trade-offs and decisions that govern their everyday lives, e.g. taking options that lead to certainty that the food bought will be eaten, providing time to go to an additional evening employment, reducing the stress of affording the energy (gas, electricity) needed to cook. These are all essential components of people’s decision-making.

One participant in one of our shopping basket workshops explained:

‘*I just get stuff that the kids will eat, because at the end of the day I can’t spend hours cooking and they don’t eat it. I want them to be fed and happy … if you don’t have a family, you can get whatever you want. If it was up to me, I would have lots of vegetables and I would go on a diet. But I don’t have time or energy to cook separate foods for myself, so I eat what my kids eat and here I am!’* [Referring to weight.]

Another participant in a shopping basket workshop told us:

‘*We would like to change everything (fresh fruit, veg, proper home-cooked-from-scratch meals) but a lot of the time you can’t, they just won’t eat it.’* [Referring to getting children to eat vegetables.]

The above examples are just some illustrations of why addressing social determinants of health, such as lack of a healthy diet, is complex. Even the term ‘healthy diet’ is open to interpretation, with cultural health just as important as physiological health for driving well-being [51]. Barriers to consuming a healthy diet are often cited as individual factors, e.g. lack of time, lack of skills, lack of confidence to try new foods, risk aversion to ‘wasting’ budget on things that might end up being thrown in the bin, wishing to avoid arguments over food [26]. However, structural and policy factors are much more influential in shaping food behaviours [52–54]. Such factors include food advertising and school food education, eligibility criteria for 'healthy start' (https://www.healthystart.nhs.uk/) vouchers and free school meals, food procurement, supermarket price fixing and farmer subsidies and income in line with cost of living (see [55,56]). Inequalities relating to health and diet are primarily driven by these societal and policy structures, which in turn impact on individuals through poverty, marginalization or exclusion [26].

### (b) Common themes for effective community engagement

The four geographical communities in FoodSEqual have used different methods to work with residents and explore the changes that they would like to see in the food systems operating in their local areas. Below we summarize the guiding principles for effective community engagement that emerged in response to the questions listed in §2c:

#### (i) Building trust and relationships

#### Existing networks and partnerships

Many successful initiatives leveraged pre-existing relationships with community organizations or Voluntary Community Social Enterprises (VCSEs). Examples include the University of Sussex’s collaboration with the Brighton and Hove Food Partnership and the University of Reading’s partnership with the Whitley Community Development Association. Plymouth academics had a long-standing track record of working with the Food Plymouth partnership and Plymouth Food Aid Network and local faith-based VCSEs, which ran food projects. The team in Tower Hamlets had few relationships to build on in St Georges Estate in Shadwell—the area where they conducted much of their research—although they did have a rich history of doing research on food with the Women’s Environment Network (WEN) who provided the initial personal contacts to gain access. The work in St Georges reflects what would be a representative starting point for many disadvantaged communities in the UK, where there is little pre-existing base for engagement.

#### Time and effort to build trust

Establishing trust with community members takes time. Plymouth’s regular face-to-face breakfast meetings and community events like cream teas were pivotal in building relationships between researchers and residents and breaking down barriers between them—such as perceptions of class or race—as was a big Tower Hamlets Food Lives event during the first year of the research. However, it should also be noted that many residents are happy to engage on a transactional basis, e.g. exchanging their knowledge in exchange for a supermarket voucher. The depth of trust and relationship between residents and researchers was therefore quite variable and often dependent on factors such as whether the researcher was of the same geographical community or cultural heritage, whether they had an affiliation with a known VCSE organization, or how challenging the request for information was.

#### Cultural sensitivity and understanding

In Tower Hamlets, understanding cultural food practices and engaging bilingual researchers helped foster connections with diverse communities. Given the culturally, racially and ethnically diverse nature of the residents they were working with, the CFRs in Tower Hamlets took it upon themselves to learn about the different food practices and food traditions of the racialized and ethnicized groups with which they work. This ensured they could have culturally sensitive conversations with potential participants for a range of research methods. This led to some valuable insights that in turn set directions for future research, e.g. understanding how the Bangladeshi community use ghee dispelled some myths about its role in causing metabolic ill-health and has led to a sub-project to consider how food messaging around oils and fats could be made more culturally appropriate [57].

#### (ii) Participatory and inclusive methods

#### Co-design and co-production

Workshops and other methods were co-designed to reflect community priorities, as seen in Brighton’s FoodSEqual policy workshops, Food Diaries in Tower Hamlets and creative engagement of school students in Whitleigh, Plymouth. Knowledge, power and inclusivity and how they affect research are acknowledged as key principles influencing co-production across TUKFS projects [27].

#### Engagement through action

Activities like cooking demonstrations, action film making (Plymouth), food photography and community markets were used to help to recruit and retain participants for our interventions and to provide spaces for people to tell us about food in their lives. Research was made more inclusive through the use of creative and accessible methods. For example, tools such as food cards, hands-on workshops, photo-voice and participatory data collection and analysis made research more inclusive and provided an equitable platform that accounted for differences in literacy levels and contribution preferences ([58]; https://online.fliphtml5.com/vvvjt/mwjl/#p=10).

#### Imagining what is not already there

People from all walks of life can struggle with conceptualizing the future and we have used a wide variety of creative methods in FoodSEqual to develop new aspects of the food system. Methods such as ‘the shopping basket’, which have incorporated techniques such as visioning, collage and zine, provided people with the opportunity to think about what they really wanted from their food system.

#### (iii) Importance of local gatekeepers and community food researchers (CFRs)

#### Role of gatekeepers

Local leaders and organizations were instrumental in bridging the gap between academics and residents. For instance, Food Plymouth acted as a mediator in Plymouth, while in Tower Hamlets, local gatekeepers helped navigate complex community dynamics. In some areas, the staffing of the FoodSEqual project reflects the practical application of this ‘bridge’, e.g. a member of the Plymouth Food Partnership is the community research manager for that location; members of the Brighton and Hove and the Plymouth Food Partnerships are community food researcher coordinators in their areas, which helped to facilitate relationships between researchers and residents.

#### Empowering CFRs

Community researchers were given additional training, such as safeguarding, that helped to integrate them into the academic world of researchand enabled them to conduct their work with the knowledge that they had the practical support of experienced academics and community development professionals. CFRs were active participants at academic conferences (within and outside the UK), at food partnership events and at consortium meetings. They were supported to have speaking roles at these events by the academic team, who also provided advocacy to emphasize the value of having the CFRs' voices heard. CFRs are co-authors on research publications (including this one) and have written blogs and policy briefs in their own voices that are published on the FoodSEqual website (https://research.reading.ac.uk/food-systems-equality/). The training provided to CFRs increased the depth of their data collection and fostered their confidence [59].

#### (iv) Flexible and context-specific approaches

#### Tailoring engagement

Different communities required different approaches. For example, informal conversations worked well in Reading, while events like foraging walks in Plymouth encouraged participation by residents. Participants in Brighton and Hove were motivated to participate in research by connecting activities to community growing projects, whereas developing a food walking tour of Shadwell that brought to life its food histories has been influential in engaging residents with different spaces and types of media in that area [60] (see also [57]; https://www.wen.org.uk/2024/03/13/food-lives-podcast/).

#### Adaptability

Responding to feedback and refining methods ensured relevance and effectiveness. Plymouth’s CFRs adjusted workshops based on trial runs and community input. In Tower Hamlets, CFRs participated in the proposed research methods themselves so that they were able to allay concerns and answer questions from residents. This was game-changing for methods such as ‘cookalongs’, where residents invited CFRs and their cameras into their homes.

#### (v) Tangible benefits for communities

#### Direct benefits

Providing vouchers, training opportunities or direct services (such as cooking classes) increased engagement and demonstrated reciprocity, i.e. researchers did not expect residents to donate their time, knowledge and skills to the research for free. These points illustrate that it is possible to have effective community engagement without necessarily relying on 1 : 1 trusted relationships being developed. Some residents are perfectly happy to engage in a transactional relationship, whereby knowledge and information are exchanged for something tangible that improves the life of that individual. In many ways, this form of engagement mitigates the risks of being seen to conduct extractive research, which can happen if researchers build cozy personal relationships to obtain their research findings before disappearing from the lives of those they befriended.

#### Feedback loops

Communities valued knowing the outcomes of their contributions. Transparent communication about research findings reinforced trust and made it more likely that residents would re-engage. Feedback sometimes took the form of an enjoyable event (https://research.reading.ac.uk/food-systems-equality/fun-with-food-whitley/), or even as a ‘curriculum enrichment’ session for school children who contribute to the research (https://research.reading.ac.uk/food-systems-equality/dinner-takes-all/).

#### (vi) Challenges and ethical considerations

#### Avoiding exploitation

Researchers emphasized the need for adequate budgets to ensure fair compensation and to avoid exploiting participants. For residents who are already coping with complex lives, economic hardship and time constraints, we had a philosophy across the project that no resident would be expected to give up their time for free. This was both to ensure that responsible research ethics were at the core of FoodSEqual, but also to demonstrate to residents how much the academic teams valued the knowledge, lived experience and solution-based thinking that residents contributed to our research.

#### Dynamic and complex communities

Engagement strategies must account for both the evolving nature of communities and the shifting political and economic landscapes within which our research was conducted. In Tower Hamlets, this was reflected in a change of strategy for some research activities where large participant numbers were needed for a single piece of work, utilizing pre-existing relationships between the charity leaders in community and sustainable food co-ops in Limehouse Town Hall and the Teviot Estate. An agility in responding to emerging needs is helpful. For example, some currently ongoing work maps and attempts to explain the gluts and shortages in the fruit and vegetable supply entering the food aid sector in recent months.

#### (vii) Key success factors

#### Leadership and team ethos

Strong leadership in each geographical area and a supportive team culture were frequently cited as enablers of effective engagement. In an environment where there is so much that could be done, there is a requirement for leaders who are able to navigate decision-making in an inclusive way so that the resource available is deployed most effectively in that area.

#### Long-term commitment

Sustained engagement and visible commitment were essential for building lasting relationships and trust. This puts pressure on project leadership to obtain legacy funding for the research that we do in the areas where FoodSEqual works. The success of our community engagement is in part because residents find our approaches relevant, interesting and/or able to provide them with a tangible benefit.

### (c) Creating social cohesion through a place-based approach

The Organisation for Economic Co-operation and Development (OECD) defines a cohesive society as one that

‘…works towards the well-being of all its members, fights exclusion and marginalization, creates a sense of belonging, promotes trust, and offers its members the opportunity of upward social mobility’

The Labonte model of health inequalities [6] includes psycho-social factors alongside the wider determinates of health; psycho-social factors include social support and social network. As such, social cohesion is an important determinate of health [61] and our sense of being part of a community is vital as a building block of health [62]. The FoodSEqual-Health Fresh Street Community intervention is a complex, multi-component intervention that actively encourages social cohesion and social connection with others by enabling the sharing of vouchers with neighbours, family and friends, and even with strangers at the till. The location of the intervention and the delivery of other activities or services alongside the Fruit and vegetable provision has developed social connections and conversations between residents in a way that is not observed when people get their food from large retailers or online. Residents are encouraged to shop for one another or to pick up another household’s vouchers if they cannot physically come to the stall themselves. This is based on trust; we do not ask people to prove who they are or why they are shopping or collecting vouchers on another person's behalf. Our intervention has highlighted the need to physically bring people together to overcome the loneliness and isolation experienced by many adults and to increase a sense of belonging [63,64].

Social cohesion is enabled by having a physical space that routinely provides a safe location for conversations and activities that, in turn, both enable a sense of social connectivity to develop and promote feelings of belonging [65,66]. In Reading, where a community development association was already established in a Council-owned building, this created opportunities for people to come together. The pop-up fruit and vegetable stall is a point of contact for multiple social and healthcare services, and the café is a place where people can not only sit and share a drink, but also connect with WCDA staff, find out more about what is happening in the local area and meet voluntary sector and public sector organization staff in a space away from their own homes. The stall catalysed engagement from groups that had not previously engaged with WCDA; a Nepalese resident of Whitley, recognized as a faith leader, was instrumental in engaging their community with the intervention and broadening the racial and ethnic diversity of residents who now routinely visit the hub. The function of a physical community space as a vehicle to enable residents to engage with food interventions was also evident in some parts of Tower Hamlets in the ‘Bridging the Gap’ project (https://www.sustainweb.org/news/nov23-bridging-gap-tower-hamlets-coop-pilot-organic/), which based its food co-operatives in Limehouse Town Hall (https://www.limehousetownhall.co.uk/mission/) and The Teviot Centre (https://www.leadersincommunity.org/the-teviot-centre/), both of which were already hubs for a range of community-led events. In Plymouth and in Brighton and Hove some physical spaces existed, e.g. the Salvation Army base and a Community Kitchen, respectively, but they were associated with one-off events rather than as spaces where people routinely gathered to participate in activities together. By contrast, there was no community space in St George’s Estate in Tower Hamlets. Interestingly, in Plymouth, participants in the Fresh Street-Community intervention voiced that they were pleased that the project ran in a different venue frrom the local community hub because they associate the latter with stigma and shame because the food bank was located there.

### (d) Engaging as equal partners

We had to change our thinking as we progressed the FoodSEqual project: we set out to innovate new products, new supply chains and new policies as separate exercises but we have instead found that scenarios developed with the community and other stakeholders in each location actually are an interaction of the three elements. For example, the Plymouth Fish Finger [33] emerged as a product development opportunity in response to residents articulating that they wanted to use local fish and reduce waste. However, the product needs new school food procurement policy, investment in equipment to enable scale-up of production and new supply chains to make it commercially viable and best able to meet the needs identified by the community themselves. Similarly, growing culturally appropriate vegetables in the UK to supply Tower Hamlets residents with more sustainable, healthy and fresher produce fulfils a role of connecting people with their heritage. Our work includes the integration of other disciplines such as supply chain innovation and plant breeding to allow the intervention to be successfu

The close working between residents and food industry partners was initiated at the start of FoodSEqual, before we had any concept of the sub-projects illustrated in the paragraph above. This long-term approach to enabling different types of stakeholders to work together has been key to success, since we tackled early-on some of the issues around trust and power dynamics that some residents associated with the food industry. On several occasions we were able to bring community food researchers and/or residents and food industry representatives together with Universities, which were able to provide independent coordination and neutral venues to facilitate discussions. In FoodSEqual, we worked through progressively deeper engagement, starting with very open ‘show and tell’ sessions that revealed food industry representatives as being just as human as the residents in the same room in terms of experiencing complex motivations for the food they personally consume. We developed a video to illustrate to residents how industry typically undertakes new product development, highlighting the considerations and decisions facing a business that go far beyond recipe development. Community Food Researchers were invited to visit a range of food businesses, which was hugely powerful in breaking down mistrust and connecting people with how food reaches their plate. Our Advisory Board includes people with a long history of working in large food industries and their participation in all-team project meetings and bespoke contributions to particular aspects of product development have demonstrated to residents that industry can be part of the food systems solution. Any initial mistrust has been replaced by excitement that food products which residents helped to develop may find their way into a local store or school where people like them will be able to consume it.

Because of their lived experience, residents very often have the skills and expertise to articulate the change they want to see and the solutions that would work but they lack resources and access to power and networks, leaving people unable to enact them [67]. A community-centred approach seeks to address this by disrupting traditional decision-making systems, directly connecting the community with the producers or service providers (Community Participatory Action Research 2021−2022: see [68]). However, the food industries supplying that area, the local authorities making policy decisions or the businesses retailing food all are configured to operate at a much bigger scale. It is a challenge to develop solutions that are appropriate at the micro-scale of a community, whilst still having value within a local authority plan or providing a return on investment from a business that must answer to its shareholders. Many of the barriers to enabling a local food system to be developed at scale were highlighted in the recent report on [69]. The concept of co-development has to be taken a stage further, perhaps using the Return on Social Investment Framework as a guide [70] and conceptualizing ‘local food’ as a system that brings closer connections between producer, processor and eater and maximizes the economic and environmental benefits in the area where the food is produced and sold [69].

### (e) Meeting aspiration through intervention

Our benchmarking in FoodSEqual-Health established that fruit and vegetable consumption was lower in the two participating communities (Plymouth and Reading) than the NDNS data report for the most deprived decile in its records. The UK ‘Five-a-day’ campaign has yielded an increase of only half a portion per person per day over the 10 years of its existence [71]. In line with the Food Standards Agency poll [72], in which 68% of people said they would like to eat more fruit and vegetables, our communities have consistently told us that there is no shortage of aspiration to consume more fruit and vegetables.

Policy workshop participants

‘*It’s good to be eating healthy and it would be nice if you can go to a shop and afford all those nice, healthy looking vegetables… buy that good quality stuff. I wouldn’t be able to afford to do it.’*

*‘You need 5 a-day, don’t you? [BUT] you’ve got to have the [FUNDS] money-wise to buy the stuff, yes.’*

Fruit and veg workshop participant

‘*One of the best things about the surplus is the fruit and veg. We all want to eat them but you know how expensive they are. It’s healthy. It’s what we want.’*

Shopping Basket workshop participant

‘*If you’ve got like two quid and you’re going into Morrisons, you’re going to grab something like something to snack on for lunch or whatever, you’re going to grab some biscuits and a pack of crisps. You buy a cheap snack. A pack of strawberries is your £2 gone.’*

People want fruit and vegetables to be good quality, culturally appropriate, safe (as in with reduced pesticide use in its production), locally available and affordable. These last two aspects led us to design FoodSEqual-Health, using the existing Fresh Street intervention as its base, but building in a strong community aspect through basing the intervention at a community centre and using local providers (wholesalers) for the fruit and vegetables. Providing vouchers is a type of cash-first intervention that choice-edits what consumers can buy with them.

After 12 months of running the Fresh Street Community we can demonstrate an increase in consumption of fruit and vegetables compared with baseline in both areas of the intervention (in Reading, the average consumption increased by 1.2 portions per day; in Plymouth, consumption increased by 1 portion per day in Fresh Street Community participants). What is perhaps more striking is the value of the community cohesion aspect of the intervention: neighbours who were ‘strangers at the till’ now visit the stall together; requests for culturally appropriate vegetables to be available have led to the engagement of a wider ethnic diversity making use of the co-located healthcare, social care and café facilities.

### (f) Policy and food partnerships as enablers of change

The concept of policy was quite new to many participants, but our co-designed policy research methods enabled residents to discover that they did interact with a wide range of policies on a daily basis, whether it was those behind the school meals framework, food production, food advertising practices or determining eligibility for healthy start vouchers [55,56]. Going beyond generating a unified voice of or for the community, in our policy workshops, we directly engaged with residents experiencing food inequalities. The lived experience of our research participants led them to initially believe that policy would always be ‘done to’ them, but FoodSEqual research has successfully engaged residents from Brighton and Hove in thinking about and contributing to policy solutions. For example, in Brighton and Hove, in collaboration with local authorities, research participants’ input has been taken further for the development a Digital Bus Stop Advert for Healthy Start Vouchers, bringing together two key policy areas and needs identified in our workshops. Our work has also been pivotal in informing local food policy strategies. The Brighton & Hove City Food Strategy is due for a refresh in 2025—a process managed by the Brighton & Hove Food Partnership. University and Community Researchers from FoodSEqual co-produced a policy briefing [55] and presented findings at the ‘Research and Evidence Review’ day (https://www.sussex.ac.uk/broadcast/read/64792), providing input towards the city’s food strategy and action plan that is currently being developed. In Reading, a policy brief has also been co-produced with Whitley Community Researchers [56] and communicated in local food policy events co-organized by FoodSEqual and Reading Borough council to help shape Reading’s Food Partnership and Food Strategy developments.

The presence of an independent organization that is seen to represent the needs of a community is seen in a positive light by many residents. In Brighton and Hove, the Food Partnership (https://bhfood.org.uk) brokers the relationship between residents and local authority. Notably, all of the communities associated with FoodSEqual had food partnerships at the outset (https://foodplymouth.org; https://www.wen.org.uk/foodpartnership/) with the exception of Reading but, drawing on the expertise of FoodSEqual and many other voluntary partners, the town launched its first ever food partnership in 2024 (https://www.reading.gov.uk/adult-care/public-health-and-staying-well/eating-well/) and is currently working towards the development of its Food Action Plan in collaboration with FoodSEqual. In areas where food partnerships are more established, they have co-developed clear strategies for their region with relevant stakeholders and residents. For example, the Food Plymouth action plan (https://foodplymouth.org/plymouth-food-action-plan-2023-2026/) has a clear articulation of what success looks like for each area, who is responsible and when it will be delivered. The city council co-developed, and are embedded in the plan, and the presence of a food partnership champion in the council ensures impact across a range of teams and locations, in common with most of the national food partnerships (Sustainable Food Places, https://www.sustainablefoodplaces.org/about/what-are-sustainable-food-places/). The evidence from food partnerships illustrates that tackling health inequalities requires systemic structural changes, but supported by individual advocates who act as ‘policy entrepreneurs’ [73] and who can provide a trusted local contact point.

## 4. Concluding thoughts

During FoodSEqual-Health we have tackled just one of the social determinants of health, that of physical and economic access to a healthy diet. The intervention was made possible by the engagement methods we had already established through FoodSEqual—we were known by our communities who had evidence that we would listen to their views and value their experiences and knowledge. We had also established the aspirations of residents and knew that access to affordable fresh fruit and vegetables was high on their priority list. We had recruited and trained community researchers, both in the principles of a community-centred approach, in undertaking participatory methods and in co-designing methods. The community researchers are the focal point around which the research can succeed; we co-develop the interventions with them as much as possible, aiming for the logistics of the intervention, the language used to describe it and to frame our questions, the methods of data gathering, the outputs we (co-)produce and the way in which we feedback outcomes to everyone to be accessible and inclusive. This takes time and was initially frustrating for researchers not used to working in this way. However, the quality of the data that the community food researchers obtain and the engagement they can generate with residents are outstanding, providing contextualized narrative alongside quantitative data that start to answer questions of ‘why’ as well as ‘what’ or ‘how’ people eat what they do. They are the lynchpin in being able to reach communities who are classified as ‘hard to reach’; co-developing the whole process with CFRs enables them to be advocates for the academic researchers during recruitment to interventions. Critically, the CFRs are able to represent the views of residents to researchers, food industry leaders and local authority decision-makers who do not often hear these voices.

In developing the Fresh Street-Community intervention used in FoodSEqual-Health we were able to draw on the experiences of running similar feasibility studies in five other areas of the UK. Through sharing these experiences, we had insight into what could work logistically, but needed to adapt Fresh Street for each of the areas implementing it according to local need, and to implement the ‘community’ aspect of the intervention for the first time. This was a good example of how an intervention (or indeed any kind of new food system-related change) needs to be locally adaptable and agile: our fruit and vegetable wholesaler partners had different business models that they could use to work with us; residents wanted slightly different things regarding adjunct health and well-being support; the spaces available for community activities were at different stages of establishment as hubs for activities associated with tackling health inequalities. However, even across two sites using different models of delivery, the provision of affordable fruit and vegetables for the community led to common findings [44]. A common approach to process evaluation means we can distinguish local nuances from core findings that will be more widely applicable across the UK food system. Residents are not the only stakeholders who participate in the evaluation; we are able to assess the potential of continuing Fresh Street-Community as part of ‘business as usual’ in the area to contribute to economic development for the wholesale businesses and employees associated with delivering the service. Community health and social care providers use Fresh Street as a magnet for residents to become engaged with healthcare; we provide evidence to local authorities outlining the value for money of Fresh Street versus the cost of providing critical care services. Through evidencing outcomes of Fresh Street-Community it has already become apparent that the benefits are not just for the quality of residents’ diet or their physical health. The ‘community’ aspect of the intervention that evidences bringing people together on a regular basis, developing interpersonal connections and care for others within the community is proving to be one of the most influential legacies of the study.

Addressing food system determinates of health inequalities requires a holistic approach and a recognition of the wider/social determinants of health that are the cause of health inequalities. To date, we have developed novel, sustainable fish products and increased access (physically and economically) to culturally appropriate fresh fruit and vegetables. Both fish and fresh fruit and vegetables are consumed at below the levels of government dietary recommendation in population groups living with deprivation. Increasing access to both has the potential to reduce the widening health inequalities observed in society, while also recognizing the sustainability and systems nuances around their production and consumption.

One of the most exciting findings from FoodSEqual-Health and some of the FoodSEqual interventions is the growth in social cohesion we have observed. Residents who have taken part in research are empowered to tackle problems, to support one another and to work towards further developing their local food system to one that meets their needs. Residents who participated in both sites of FoodSEqual-Health interventions told us what they wanted us to do next to address their food systems challenges and these views were used to inform applications for new research grant proposals submitted to UKRI. If we are to address food system determinants of health inequalities we have to include residents who are at greatest risk of health inequalities in every part of the process (Box 1). However, despite the importance of psycho-social factors, material aspects, such as access to money and resources, are still critical limitations to addressing inequalities. Affordability is by far the most common inhibitor of dietary shift.

BOX 1. **BRAVE TIPs for community engagement in research.**

**Table:** 

**Benefit now**: Provide direct benefits that recognize the time, knowledge and skills provided by participants. Ensure that participants are provided with feedback communication about how their research input made a difference.	**Empower within**: Recruit, train and empower Community Food Researchers who have deep local knowledge of the residents you want to reach.
**Resilience**: Do not back away from difficult issues—such as the prevalence of poverty, tensions between residents and local authorities or the myriad of lifestyle factors that influence what residents actually eat. These are the issues that give research an authenticity if they are incorporated into the findings.	**Time**: Take sufficient time to build networks, partnerships and trust between residents and researchers.
A messy solution that makes a positive difference is better than a neat suggestion that gets ignored.	
**Adaptable**: Be prepared to adapt methods, timing and questions for each area you work in, so they are appropriate for each local context and any shifting of economic and political landscapes that occur during the research period.	**Involve:** Engage local organizations already working in your area, take time to know gatekeepers and influencers.
**Value**: Demonstrate to residents that the research team value their lived experience, food traditions and will take time to understand the context of residents’ food lives, as described by residents themselves.	**Participatory**: Be creative and inclusive in devising research methods—and involve residents in their development every step of the way.

Participant at fruit and veg workshop on theme of pricing:

*‘I would snack on strawberries if they were the price of a pack of biscuits.’*

Systemic change, at national and local authority level, is required to place food strategy and action plans at the heart of tackling dietary determinants of health inequalities. Food Partnerships should be supported to develop locally agile solutions that enable producers, manufacturers, retailers and residents to achieve a healthy, economically viable and accessible food system in each area.

## Data Availability

School Research Ethics Committee approval were obtained for all the methods listed in this paper. The paper collates opinion drawn from these approved studies, rather than directly reporting the study results. A list of all methods covered by ethics agreements is available at [34]. Supplementary material is available online [74].
